# Extraction of Fatty Acids and Phenolics from *Mastocarpus stellatus* Using Pressurized Green Solvents

**DOI:** 10.3390/md19080453

**Published:** 2021-08-08

**Authors:** Uxía Cid, Paula Rodríguez-Seoane, Beatriz Díaz-Reinoso, Herminia Domínguez

**Affiliations:** 1 CINBIO, Centro de Investigaciones Biomédicas, Departamento de Enxeñería Química, Facultade de Ciencias, Universidade de Vigo, Campus Ourense, Edificio Politécnico, As Lagoas, 32004 Ourense, Spain; uxiacid9@gmail.com (U.C.); paurodriguez@uvigo.es (P.R.-S.); 2 CITI, Centro e Investigación, Innovación y Transferencia, Universidade de Vigo, Parque Tecnolóxico de Galicia, Rúa Galicia N° 2, 32900 Ourense, Spain

**Keywords:** *Mastocarpus stellatus*, supercritical CO_2_, subcritical water, microwave heating, omega 3, antioxidants

## Abstract

Polyunsaturated fatty acids are well known for their protective properties in relation to different skin diseases. Although seaweeds possess a low lipid fraction, they could act as an alternative renewable source of polyunsaturated fatty acids whenever other valuable seaweed components are also valorized. In this study, a biorefinery process using *Mastocarpus stellatus* as a model seaweed was proposed. The process started with the supercritical carbon dioxide extraction of the lipid and phenolic fractions. The influence of pressure during extraction with pure supercritical CO_2_ was studied while operating at a selected temperature and solvent flow rate. Kinetic data obtained during the ethanol-modified supercritical CO_2_ extraction were fitted to the spline model. Sequential processing was proposed with (i) pure CO_2_ to obtain a product with 30% PUFA content and ω-3:ω-6 ratio 1:1, (ii) ethanol-modified CO_2_ to extract phenolics, and (iii) microwave-assisted subcritical water extraction operating under previously optimized conditions for the extraction of phenolics, carrageenan and protein fractions. The composition of the supercritical extracts showed potential for use in both dietary and topical applications in skin care products. The remaining solids are suitable for the extraction of other valuable fractions.

## 1. Introduction

Long-chain fatty acids contribute to the maintenance of normal skin functions [1,2] and polyunsaturated fatty acids (PUFA), particularly omega-3 (ω-3 PUFAs), exhibit interesting properties in relation to various dermatological conditions [3,4]. Given their high safety profile, low cost, and ease of supplementation, dietary ω-3 PUFAs have been proposed as an adjunct therapy for their anti-inflammatory properties and ability to induce a protective effect against cutaneous diseases, including psoriasis [4,5] and acne vulgaris [6,7,8]. In addition, they can be topically applied to exert an anti-inflammatory effect [9] and are successful in managing psoriasis [5,10] and for wound healing resolution [11,12]. Polyunsaturated fatty acids are among the bioactives from marine resources that show potential as natural skin care agents for the development of novel cosmetics and nutricosmetics with dermatologic benefits and as promising applications for skin regeneration, photoprotection and wound healing [1,13,14].

Many studies are available that show the results of using fish oil as a potential supplement to ameliorate the severity of some skin disorders [3]. However, declining fish stocks and the limitations for people who are allergic or vegetarian as well as the restricted use of animal-based ingredients in cosmetics, has led to the search for alternative marine sources for ω-3 PUFA, such as micro and macroalgae [15]. Despite their abundance, macroalgae are poorly exploited and they have a low lipid content but a high proportion of PUFAs, combined with other bioactive metabolites [16]. Low-cost biomass, such as invasive seaweeds, and algal blooms are especially promising [17]. Seaweed lipids present an interesting ω-6:ω-3 ratio, but the seaweed lipid content is low and valorization of the whole material is preferred, following a more sustainable approach based on the biorefinery concept. In addition to lipids, macroalgae also contain a number of bioactives, such as polysaccharides, phenolics, amino acids, chlorophylls, carotenoids, and sterols, which have potential benefits for human health [16], and other valuable components are found in their lipophilic fraction, particularly carotenoids and sterols. Therefore, the valorization of this fraction would offer some practical advantages, particularly in relation to the stabilization of the products. Oils containing a high amount of ω-3-PUFAs are highly susceptible to oxidation, especially during conventional extraction when they are exposed to high temperature and oxygen. Natural antioxidants have been successfully proposed to prevent and/or delay this oxidative deterioration and seaweeds could be a good source of antioxidant components [18]. 

Supercritical carbon dioxide (sc-CO_2_) extraction is selective for fats and oils, and the oxygen-free conditions are suitable for the extraction of thermosensitive compounds. In comparison with conventional extraction, the solvent is non-toxic, the duration of the process can be shortened and the selectivity can be tuned by adequate selection of the operational conditions [19,20,21]. Furthermore, the easy separation of the solvent by decompression ensures a safe solvent-free product [15]. Among the different techniques, sc-CO_2_ extraction of oil from terrestrial sources provides higher yields and the addition of ethanol as a modifier has been proposed to extract oils with the desired ω-6:ω-3 ratio [17,22,23] or with the lowest saturated fatty acids content compared to oils from commercial, cold solvent and pressing extraction [16]. The addition of ethanol as a modifier not only increases the extraction yield but also increases the total phenolics and carotenoids content as well as the antioxidant activity [17]. This would be an alternative strategy to limit oxidation of the highly susceptible PUFA [24,25,26]. The integration of this process in a biorefinery process would allow the valorization of the exhausted solids [27]. Subcritical water processing has been proposed as a green technique suitable for the extraction of protein, polysaccharides and phenolics from vegetal and algal biomass [28]. Furthermore, intensification with microwave heating has proved successful for the rapid extraction of carrageenan and phenolic compounds from *Mastocarpus stellatus* [29].

The aim of the present study was to evaluate the initial stage of scCO_2_ extraction of lipidic and phenolic fractions from *Mastocarpus stellatus*, which was used as the model alga. The remaining solids were further treated under previously optimized conditions of subcritical water processing [29] for the recovery of carrageenan, protein and phenolic fractions. The overall scheme would aid in integrating the lipid extraction stage with the further extraction of other fractions from a red seaweed, while following a biorefinery scheme for the sequential recovery of valuable fractions.

## 2. Results

### 2.1. Supercritical Fluid Extraction

#### 2.1.1. Pure Carbon Dioxide

**Figure 1:**
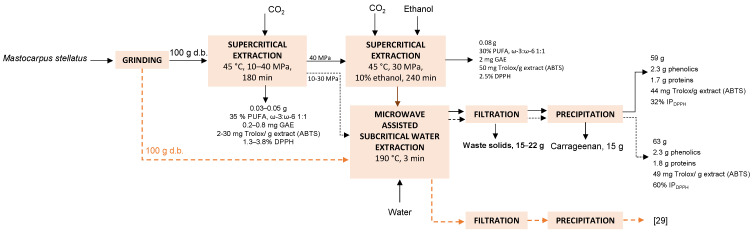
Schematic flow diagram and mass balance of the sequential process of sc-CO_2_ extraction of lipids, phenolics, carrageenan and protein from *Mastocarpus stellatus*.

The initial processing stage was performed using pure carbon dioxide to obtain the most valuable and thermosensitive components, that is, the lipidic and phenolic fractions. Further processing with ethanol modified carbon dioxide was proposed to enhance the phenolics yield and antiradical properties. The next stages were aimed at solubilizing other valuable components from the raw material by applying aqueous extraction techniques under previously optimized extraction conditions. A schematic flow diagram of the process is presented in Figure 1.

**Figure 2:**
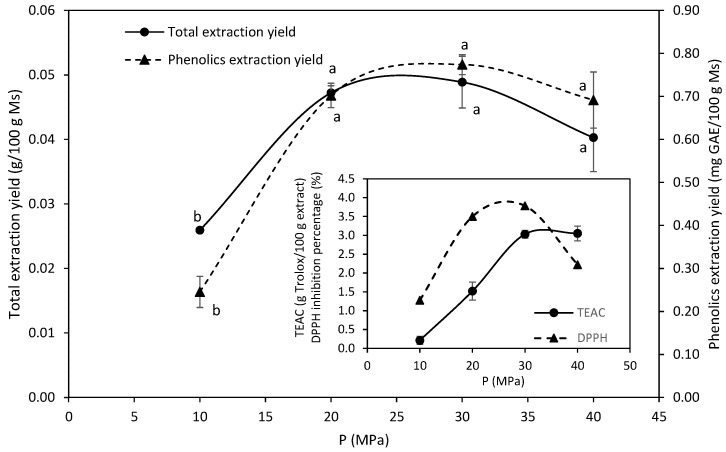
Effect of pressure at 25 g CO_2_/min, 45 °C and 3 h on the total extraction yield and total phenolics extraction yield during sc-CO_2_ extraction of *M. stellatus*. Different letters in the same series represent significant difference (*p* < 0.05). The antiradical capacity against ABTS and DPPH are also presented.

**Figure 3:**
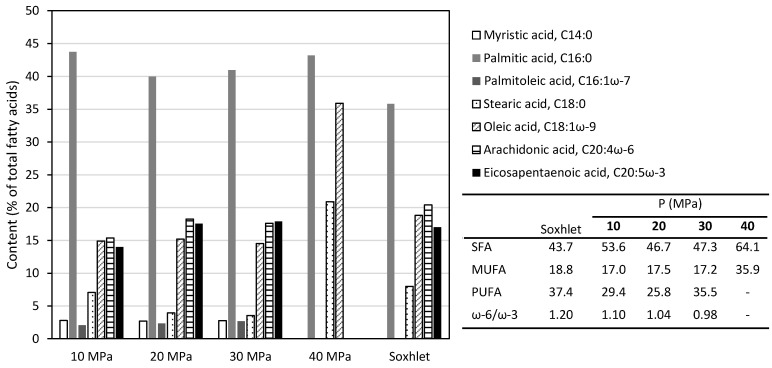
Fatty acid profile of *M. stellatus* extracts obtained with hexane (Soxhlet) and with sc-CO_2_ at different operation pressure and 45 °C for 3 h. The relative standard deviation of the data is less than 15%.

The extraction temperature was fixed at 45 °C, based on the literature data for brown seaweeds, which reported that except at low pressures, temperatures in the range 40–50 °C are favored for the extraction of lipids [21,22],and also help to avoid degradation of the phenolic fraction. The extract solubility in sc-CO_2_ is known to increase with pressure [30], and as expected, pressure strongly affected the yields (Figure 2) and type of fatty acids (Figure 3). The extraction yield reached a maximum at 30 MPa, corresponding to only 5% of the Soxhlet extractables in hexane, which provided 1.1 g/100 g seaweed. However, other authors have reported yields comparable to that attained in chloroform:methanol (2:1) for *Sargassum hemiphyllum* extracted with sc-CO_2_ at 38 MPa, 50 °C for 1 h [22]. 

The extraction of phenolic compounds was enhanced at higher pressures, with a steady increase in the range studied. As expected, the antiradical capacity against ABTS and DPPH followed a similar trend and markedly increased with pressure up to 30 MPa (Figure 2). While the ABTS radical scavenging was attained at 40 MPa (TEAC value 3.05 g Trolox/100 g extract), the inhibition percentage against DPPH radical was maximal at 30 MPa (3.78%). These activities were determined in the directly obtained extract without further concentration. Both activities were very low compared to those of synthetic antioxidants, such as BHT or BHA, which exhibited an EC_50_ of 2.8 and 0.24 g/L, respectively, against DPPH and the TEAC values were 2.1 and 1.8 g Trolox/g, respectively. Other authors have reported more potent antioxidant properties, which they ascribed to the phenolic compounds found in the supercritical extracts [31,32]. Furthermore, the possibility of obtaining synergistic beneficial effects on the skin care properties should be evaluated.

The fatty acid (FA) profile was influenced by the solvent and operation pressure during sc-CO_2_ extraction (Figure 3). Palmitic acid was the most abundant regardless of the solvent and the conditions with the content in supercritical extracts being higher than in hexane extracts. The stearic and oleic were preferentially extracted at 40 MPa and the major PUFAs in *M. stellatus* were eicosapentaenoic acid (EPA, C20:5 ω-3) and arachidonic acid (ARA, C20:4 ω-6), but they did not increase with sc-CO_2_. An opposite trend was observed for *S. hemiphyllum* sc-CO_2_ extracts compared to chloroform/methanol [22]. The SFA content decreased with pressure from 54% at 10 MPa to 47% at 30 MPa, while the PUFAs content increased from 10 to 30 MPa and were not detected at 40 MPa. A decrease in the ω-6:ω-3 ratio was observed when pressure increased from 10 to 30 MPa and the values were lower than in hexane extracts.

The beneficial effect of increasing pressure has also been reported for the extraction of PUFAs, which were favored over saturated ones from the brown seaweed *S. hemiphyllum* [22]. It has been reported that as the pressure and solvent density increases, the amount of unsaturated fatty acids and degree of unsaturation increases whereas the saturated/unsaturated and saturated/polyunsaturated ratios decrease, suggesting that triglycerides containing more unsaturated fatty acids are soluble at higher densities [30]. 

#### 2.1.2. Supercritical Extraction with Ethanol Modified CO_2_

**Figure 4:**
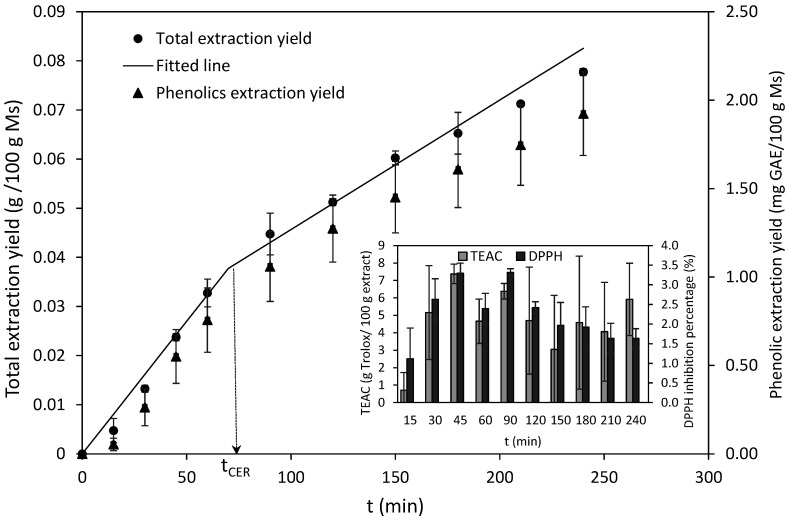
Extraction kinetics of total cumulative extractables (a fitted curve using the spline model is also included) and total phenolics, during supercritical CO_2_ extraction (30 MPa, 45 °C, 10% ethanol) of the *M. stellatus* solid phase after extraction with pure sc-CO_2_. The antiradical properties against ABTS and DPPH of each collected fraction are also presented.

The influence of the extraction time was evaluated for a period of 240 min at 45 °C and 30 MPa on the solid phase obtained after the extraction with pure sc-CO_2_. Data in Figure 4 show a similar pattern for the total extraction yield and the total phenolics extraction yield. The addition of ethanol increased the total yields and also the phenolic content in the extracts from 1.5 to 2.5 g GAE/100 g extract when compared to the stage with pure CO_2_. The latter value is comparable to that of extracts obtained by drying with microwave hydrodiffusion and gravity of the water phase [33]. The phenolic extraction yield attained in this experiment reached 1.9 mg/100 g *M. stellatus*, a value lower than that reported for other red seaweed processed under comparable conditions, i.e., *Gracilaria mammillaris* at 30 MPa, 60 °C and 8% ethanol yielded 3.8 mg GAE/100 g seaweed after 240 min [34].

The antiradical properties of the sampled fractions reached a maximum at 45 min, but the differences were not significant at times longer than 30 min. The values attained were very low, the TEAC value was less than 8 g Trolox/100 g extract and the inhibition of DPPH was less than 3% (Figure 4). However, the addition of ethanol as a cosolvent increased both the extraction yields and the radical scavenging activity of *S. muticum* by three times [17], and increased the phenolic content and antiradical properties of *Sargassum horneri* compared to conventional solvents [35]. Siahaan et al. [36] reported higher extraction yields of FA (EPA and DHA), and phenolic compounds with antiradical properties from brown seaweeds with ethanol-modified sc-CO_2_ compared to Soxhlet extraction with acetone, hexane, or methanol in a process ten times longer. The addition of cosolvent also influenced the proportion of the lipid classes.

The extraction curve showed two of the three characteristic periods of a SFE process: a constant extraction rate (CER) where solutes easily accessible by the solvent are extracted mainly by convection and a falling extraction rate (FER) period, where both convection and diffusion in the solid phase controls the mass transfer process. The diffusion-controlled (DC) period, where mass transfer is controlled only by diffusion, was not reached during the process time used. The lack of the DC period line indicates that the raw material was not exhausted after 240 min of extraction. The total extraction yield was modeled according to a spline model following the method described by Meireles [37]. The fitting was used to estimate the kinetic parameters for the CER period (Table 1): the time (t_CER_), the mass transfer rate (M_CER_), the mass ratio of the solute in the supercritical phase (Y_CER_) and the yield achieved in this period (x_CER_). The model shows an acceptable prediction of the experimental data (Figure 4). The yield at the end of the CER period (70.01 min) was 0.038%. According to Pereira and Meireles [38], about 50% to 90% of total yield can be extracted during the CER period, which underlines the relevance of this first period in terms of scale-up and process design [38].

**Figure 5:**
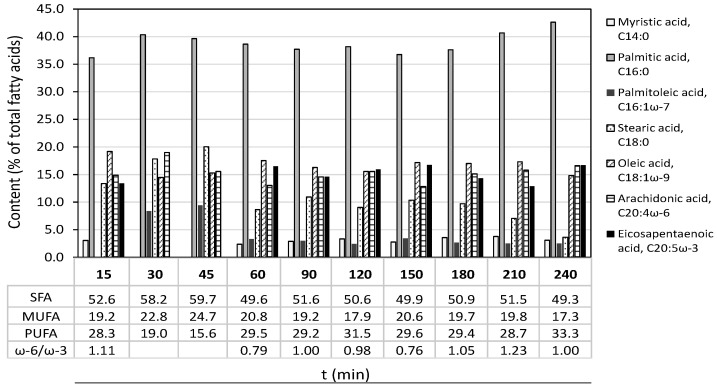
Extraction kinetics of fatty acid profile of each collected fraction during supercritical CO_2_ extraction of *M. stellatus* at 30 MPa, 45 °C with 10% ethanol. SFA: Saturated fatty acids; MUFA: Monounsaturated fatty acids; PUFA: polyunsaturated fatty acids.

Data in Figure 5 show that the relative content of the major FA groups was quite well maintained during the extraction period at 30 MPa, 45 °C using 10% ethanol. However, a slight reduction in the proportion of stearic, oleic and palmitoleic acids and an increase in palmitic acid was noticed. Palmitic acid was the major fatty acid collected during the extraction process, followed by oleic acid and the PUFAs, EPA and arachidonic acid (AA, C20:4 ω-6). The ω-6:ω-3 ratio ranged from 0.76 to 1.2. The high palmitic acid content suggests it could be suitable for cosmetic use, and some fatty acids can be effective and safe permeation enhancers for transdermal drug delivery through the skin [39,40] or for promoting skin permeability through lipid fluidization within the stratum corneum [41].

They are also attractive for cosmetic applications due to the bioavailability of eicosapentaenoic acid (EPA, 20:5, ω-3) and docosahexaenoic acid (DHA, 22:6, ω-6) to keratinocytes and fibroblasts and their ability to inhibit ultraviolet B-induced skin inflammation [42]. The topical use of DHA can also accelerate the healing of skin wounds in vivo through, among other mechanisms, the modulation of inflammatory activity [43].

### 2.2. Microwave-Assisted Subcritical Water Extraction

The residual solids remaining after the sequential extraction with pure and with ethanol-modified CO_2_ were processed by microwave-assisted subcritical water operating under previously selected conditions for maximizing the extraction of phenolics, protein, sulfate and the antiradical properties of the extracts. Whereas the hybrid carrageenan fraction obtained at 150–170 °C presented adequate biopolymer features, the carrageenan solubilized at 190 °C did not produce gels or higher polymer content or ionic strength would be required to observe them [29]. However, carrageenan oligosaccharides have been obtained under these suboptimal conditions for both yield and the viscoelastic behavior of carrageenan hydrogels (unpublished data), and these compounds have other promising biological properties, such as antitumoral or antiviral (unpublished data).

The extraction yield in aqueous media was not influenced by the previous sc-CO_2_ extraction, similarly, the phenolic and protein content of the extracts were almost unaffected compared to the direct subcritical water processing of the seaweed [29]. However, the antiradical activity against DPPH was reduced if a previous sc-CO_2_ with ethanol was applied before the aqueous extraction.

The sequential combination of stages could provide other commercially interesting products in addition to the ω-3 enriched oil obtained both with pure and with ethanol modified sc-CO_2_. The protein can be extracted under these conditions, but it may be hydrolyzed into peptides and may also contribute to the antioxidant properties, as well as the soluble carrageenan oligomers in the aqueous phase. These fractions are known to offer interesting biological properties for skin applications and should be evaluated in future studies.

An advantage of using a “green” extraction solvent is the opportunity it provides to valorize the residue or a co-product that is soluble in sc-CO_2_ [20]. Other red seaweed biorefinery approaches to valorizing the lipid fractions have been reported [27,41,44]. Peñuela et al. [45] proposed an enzyme-assisted extraction of a water-soluble extract rich in proteins and sulfated polysaccharides, a lipid fraction rich in PUFA and microwave-assisted extraction of ι-carrageenan from *Solieria filiformis*. They reported a lipid yield of 0.7% using dichloromethane/methanol (7:3 *v*/*v*) for 24 h, and the FAs profile showed 74% SFAs, 17% MUFA and 14% PUFAs, containing EPA and AA and an ω-6/ω-3 ratio of 0.9. 

In summary, the initial supercritical CO_2_ extraction of the dried material allows modulation of the operational conditions to obtain a lipid fraction with an optimal ω-6:ω-3 ratio and with phenolic compounds that could confer protection against oxidation. These extracts need to be further tested for the development of novel active products for skin care and treatment. Additional studies should also be oriented to the evaluation of a similar processing scheme for seaweeds with a higher content of ω-3 PUFAs [46]. 

## 3. Materials and Methods

### 3.1. Seaweed

Dehydrated *Mastocarpus stellatus* (Ms) alga from ecological production was kindly provided by Porto Muíños (Cerceda, A Coruña, Spain). Seaweeds were milled and stored in closed plastic bags before use. Proximal composition analyses revealed that the alga contained 13% moisture, and the dry content included 18% protein, 18% ash, 2.2% ethanolic extract, 1.1% lipids and 48% carbohydrates, with galactose being the major constituent and accounting for 28% of the carbohydrate fraction.

### 3.2. Soxhlet Extraction

The total lipids were gravimetrically determined by Soxhlet extraction with hexane. In summary, 80 g of dried seaweed was placed into a cellulose extraction thimble, and extracted with 80 mL of hexane for 12 h at a temperature of 70 °C. After the extraction, the solvent was evaporated at 50 °C and 264 mbar using a vacuum evaporator.

### 3.3. Supercritical CO_2_ Extraction

The dried seaweed (100 g) was mixed with glass beads and packed into a 1 L cylinder extractor (Thar Designs SFE-1000F-2-C10, Pittsburgh, PA, USA). The CO_2_, precooled in a PolyScience bath (Niles, IL, USA, model 9506), was pumped (P-200A piston pump, Thar Design Inc., Pittsburgh, PA, USA) at 25 g CO_2_/min. The temperature in the extractor was fixed at 45 °C, a relatively high value, without affecting the thermostable components, and the pressure ranged between 10 and 40 MPa. The extract was collected in a separator operating at 25 °C. The extraction time was fixed at 240 min. The extracts were further recovered from the separator using absolute ethanol, which was then removed using a rotavapor at 50 °C and 97 mbar and the extracts were kept under N_2_, at −20 °C in the dark until analysis. 

The residual solids after extraction at 40 MPa were further extracted at 45 °C and 30 MPa with a CO_2_ flow rate of 25 g/min using absolute ethanol as a modifier, added at 10% wt. with an HPLC pump (Scientific Systems Inc., State College, PA, USA, model Series III). The extraction yield was measured at pre-established time intervals in order to study the process kinetics.

### 3.4. Microwave-Assisted Subcritical Water Extraction

The sc-CO_2_ processed seaweed was mixed with distilled water at a liquid to solids ratio (LSR) 30 (*w*:*w*) and treated in a Monowave 450 (Anton Paar, Graz, Austria) microwave reactor at 190 °C for 3 min after reaching this temperature (Figure 1). These conditions were selected according to a previous study for the maximal extraction of phenolic compounds [29]. After they were cooled down to 55 °C, the liquid and solid phases were separated by vacuum filtration through filter paper until the liquid phase was completely drained. The liquid fraction was mixed with the same volume of 96% ethanol (Sigma-Aldrich, St. Louis, MO, USA) with the aim of precipitating the solubilized carrageenan, which was then dried at 40 °C.

### 3.5. Analytical Methods

Total extraction yield was gravimetrically determined. Total phenolic content was measured according to the Folin–Ciocalteu method described by Singleton and Rosi [47] using gallic acid (Sigma Aldrich, USA) as the standard. The soluble protein content was spectrophotometrically determined using bovine serum albumin (Sigma Aldrich, USA) as the standard using the method proposed by Bradford [48]. 

The antiradical activity was determined against two widely used species. The radical scavenging capacity of DPPH (1,1-diphenyl-2-picrylhydrazil) was determined from absorbance readings at 515 nm following the method proposed by von Gadow et al. [49]. The percent reduction in the absorbance in a mixture prepared with 2 mL of a 3.6 × 10^−5^ M methanolic solution of DPPH (Fluka, Munich, Germany) and 50 mL of a methanolic solution of the extract collected in the separator was determined after 16 min at room temperature. 

ABTS (2,2′-azinobis (3-ethylbenzothiazoline-6-sulfonic acid)) radical scavenging was determined with the method described by Re et al. [50] by adding 1 mL of diluted ABTS (Sigma Aldrich, St. Louis, MO, USA) to 10 μL of extracts or Trolox (6-hydroxy-2,5,7,8-tetramethylchroman-2-carboxylic acid) (Sigma-Aldrich, USA). The mixture was incubated at 30 °C. After 6 min, absorbance was read at 734 nm, and results were expressed as the Trolox equivalent antioxidant capacity (TEAC).

### 3.6. Fatty Acid Composition

Fatty acid methyl esters (FAME) were prepared according to UNE-EN ISO 12966-3:216, and then analyzed by a GC–MS QP 2010 (Shimadzu, Kyoto, Japan). The following temperature profile was applied: 50 °C (2 min), gradient increase at 10 °C/min up to 240 °C, which was maintained for 27 min. FAMEs were identified by comparing their mass spectra with those of authentic standards and NIST MS Search 2.0 library. The average values of two independent determinations were expressed as a percentage of total FAME.

### 3.7. Statistical Analysis

Significant differences between results were calculated by analysis of variance (ANOVA) using the MINITAB 19 software (State College, PA, USA). The significant differences (*p* < 0.05) were evaluated by Tukey’s test. Mean values and their standard deviations were calculated and presented on the figure as error bars.

## 4. Conclusions

In this study, a sequence of pressurized green solvent extraction for the biorefinery of *Mastocarpus stellatus* was proposed. The initial supercritical CO_2_ extraction of the dried material allows the modulation of the operational conditions to obtain a lipid fraction with an optimal ω-6:ω-3 ratio and with phenolic compounds. However, the extraction yields obtained in the present study were lower than expected according to the literature and more intense grinding and prolonged extraction times are probably required. Other valuable seaweed components, carrageenan oligomers, protein and peptides, and phenolic compounds could also be obtained following sequential processing using green solvents. This multistage operation represents a sustainable scheme for the integral utilization of seaweed resources using non-toxic renewable solvents.

## Figures and Tables

**Table 1:** Adjusted parameters of the spline model applied to the supercritical extraction at 30 MPa, 45 °C with 10% ethanol of the *M. stellatus* solid phase after extraction with pure sc-CO_2_.

Adjusted Parameters		R^2^
**t_CER_ (min)**	70.01	0.994
**M_CER_ (g/min)**	5.39 × 10^−4^	
**Y_CER_ (g extract/g CO_2_)**	2.16 × 10^−5^	
**x_CER_ (%)**	0.038	
**S/F_CER_**	17.5	

CER: Constant extraction rate. t_CER_: Time of the end of the CER period. M_CER_: Mass transfer rate during the CER period. Y_CER_: Mass ratio of the solute in the fluid phase at the extraction vessel outlet for the CER period. x_CER_: Yield in the CER period. S/F_CER_: Solvent to feed mass ratio in the CER period.

## Data Availability

Data are contained within the article.

## References

[1] Ahmad A., Ahsan H. (2020). Lipid-based formulations in cosmeceuticals and biopharmaceuticals. Biomed. Dermatol..

[2] Yang M., Zhou M., Song L. (2020). A review of fatty acids influencing skin condition. J. Cosmet. Dermatol..

[3] Huang T.-H., Wang P.W., Yang S.C., Chou W.L., Fang J.Y. (2018). Cosmetic and Therapeutic Applications of Fish Oil’s Fatty Acids on the Skin. Mar. Drugs.

[4] Thomsen B.J., Chow E.Y., Sapijaszko M.J. (2020). The potential uses of omega-3 fatty acids in dermatology: A review. J. Cutan. Med. Surg..

[5] Afifi L., Danesh M.J., Lee K.M., Beroukhim K., Farahnik B., Ahn R.S., Yan D., Singh R.K., Nakamura M., Koo J. (2017). Dietary Behaviors in Psoriasis: Patient-Reported Outcomes from a U.S. National Survey. Dermatol. Ther..

[6] Jung J.Y., Kwon H.H., Hong J.S., Yoon J.Y., Park M.S., Jang M.Y., Suh D.H. (2014). Effect of dietary supplementation with omega-3 fatty acid and gamma-linolenic acid on acne vulgaris: A randomised, double-blind, controlled trial. Acta Derm. Venereol..

[7] Melnik B.C. (2015). Linking diet to acne metabolomics, inflammation, and comedogenesis: An update. Clin. Cosmet. Investig. Dermatol..

[8] Aslan I., Ozcan F., Karaarslan T., Kirac E., Aslan M. (2017). Decreased eicosapentaenoic acid levels in acne vulgaris reveals the presence of a proinflammatory state. Prostaglandins Other Lipid Mediat..

[9] Ames F.Q., Bracht L., Sato F., Vizioli L., Ambrósio B., Oliveira L.A.D., Parreira de Lima E., Kenji Nakamura Cuman R., Luciano Baesso M., Aparecida Bersani-Amado C. (2020). Fish oil preparation inhibits leukocyte recruitment and bands that characterize inflamed tissue in a model of phenol-induced skin inflammation: Percutaneous penetration of a topically applied preparation demonstrated by photoacoustic spectroscopy. Nat. Prod. Res..

[10] Upala S., Yong W.C., Theparee T., Sanguankeo A. (2017). Effect of omega-3 fatty acids on disease severity in patients with psoriasis: A systematic review. Int. J. Rheum. Dis..

[11] Jara C.P., Mendes N.F., Prado T.P.D., de Araújo E.P. (2020). Bioactive Fatty Acids in the Resolution of Chronic Inflammation in Skin Wounds. Adv. Wound Care.

[12] Dorweiler B., Trinh T.T., Dünschede F., Vahl C.F., Debus E.S., Storck M., Diener H. (2018). The marine Omega3 wound matrix for treatment of complicated wounds: A multicenter experience report. Gefasschirurgie.

[13] Kim S.-K. (2014). Marine cosmeceuticals. J. Cosm. Dermat..

[14] Naser W. (2021). The cosmetic effects of various natural biofunctional ingredients against skin aging: A review. Int. J. Appl. Pharm..

[15] Rubio-Rodríguez N., Beltrán S., Jaime I., de Diego S.M., Sanz M.T., Carballido J.R. (2010). Production of omega-3 polyunsaturated fatty acid concentrates: A review. Innov. Food Sci. Emerg. Technol..

[16] Pereira H., Barreira L., Figueiredo F., Custódio L., Vizetto-Duarte C., Polo C., Rešek E., Engelen A., Varela J. (2012). Polyunsaturated fatty acids of marine macroalgae: Potential for nutritional and pharmaceutical applications. Mar. Drugs.

[17] Conde E., Moure A., Domínguez H. (2015). Supercritical CO_2_ extraction of fatty acids, phenolics and fucoxanthin from freeze-dried *Sargassum muticum*. J. Appl. Phycol..

[18] Balboa E.M., Conde E., Moure A., Falqué E., Domínguez H. (2013). *In vitro* antioxidant properties of crude extracts and compounds from brown algae. Food Chem..

[19] Machmudah S., Wahyu D., Kanda H., Goto M. (2018). Supercritical fluids extraction of valuable compounds from algae: Future perspectives and challenges. Eng. J..

[20] Crampon C., Boutin O., Badens E. (2011). Supercritical carbon dioxide extraction of molecules of interest from microalgae and seaweeds. Ind. Eng. Chem. Res..

[21] Cruz P.N., Fetzer D.L., do Amaral W., de Andrade E.F., Corazza M.L., Masson M.L. (2019). Antioxidant activity and fatty acid profile of yacon leaves extracts obtained by supercritical CO_2_ + ethanol solvent. J. Supercrit. Fluids.

[22] Cheung P.C.K., Leung A.Y.H., Ang P.O. (1998). Comparison of supercritical carbon dioxide and soxhlet extraction of lipids from a brown seaweed, *Sargassum hemiphyllum* (Turn) C. Ag. J. Agric. Food Chem..

[23] Devi V., Khanam S. (2019). Optimization of the ratio of ω-6 linoleic and ω-3 α-linolenic fatty acids of hemp seed oil with Jackknife and Bootstrap resampling. Chem. Prod. Process Model..

[24] Fernandes S.S., Tonato D., Mazutti M.A., de Abreu B.R., da Costa Cabrera D., D’Oca C.D.R.M., Prentice-Hernández C., Salas-Mellado M.D.L.M. (2019). Yield and quality of chia oil extracted via different methods. J. Food Eng..

[25] Sabeena Farvin K.H., Jacobsen C. (2012). New natural antioxidants for protecting omega-3 rich products. Lipid Technol..

[26] Dellarosa N., Laghi L., Martinsdóttir E., Jónsdóttir R., Sveinsdóttir K. (2015). Enrichment of convenience seafood with omega-3 and seaweed extracts: Effect on lipid oxidation. LWT-Food Sci. Technol..

[27] Rudke A.R., de Andrade C.J., Ferreira S.R.S. (2020). *Kappaphycus alvarezii* macroalgae: An unexplored and valuable biomass for green biorefinery conversion. Trends Food Sci. Technol..

[28] Álvarez-Viñas M., Rodríguez-Seoane P., Flórez-Fernández N., Torres M.D., Díaz-Reinoso B., Moure A., Domínguez H. (2020). Subcritical water for the extraction and hydrolysis of protein and other fractions in biorefineries from agro-food wastes and algae: A review. Food Bioproc. Tech..

[29] Ponthier E., Domínguez H., Torres M.D. (2020). The microwave assisted extraction sway on the features of antioxidant compounds and gelling biopolymers from *Mastocarpus stellatus*. Algal Res..

[30] Cheung P.C.K. (1999). Temperature and pressure effects on supercritical carbon dioxide extraction of n-3 fatty acids from red seaweed. Food Chem..

[31] Rozo G., Rozo C., Puyana M., Ramos F.A., Almonacid C., Castro H. (2019). Two compounds of the Colombian algae *Hypnea musciformis* prevent oxidative damage in human low density lipoproteins LDLs. J. Func. Foods.

[32] Kumar L.R.G., Treesa Paul P., Anas K.K., Tejpal C.S., Chatterjee N.S., Anupama T.K., Geethalakshmi V., Anandan R., Jayarani R., Mathew S. (2020). Screening of effective solvents for obtaining antioxidant-rich seaweed extracts using principal component analysis. J. Food Process. Preserv..

[33] Barral-Martínez L., Flórez-Fernández N., Domínguez H., Torres M.D. (2020). Tailoring hybrid carrageenans from *Mastocarpus stellatus* red seaweed using microwave hydrodiffusion and gravity. Carbohydr. Polym..

[34] Ospina M., Castro-Vargas H.I., Parada-Alfonso F. (2017). Antioxidant capacity of Colombian seaweeds: 1. Extracts obtained from *Gracilaria mammillaris* by means of supercritical fluid extraction. J. Supercrit. Fluids.

[35] Shipeng Y., Woo H.C., Choi J.H., Park Y.B., Chun B.S. (2015). Measurement of antioxidant activities and phenolic and flavonoid contents of the brown seaweed *Sargassum horneri*: Comparison of supercritical CO_2_ and various solvent extractions. Fish. Aquat. Sci..

[36] Siahaan E.A., Pangestuti R., Chun B.S. (2020). Antioxidant activity of two edible Korean seaweed oil obtained from SC-CO_2_ and solvent extraction. E3S Web Conf..

[37] Meireles M.A.A., Martinez J.L. (2007). Extraction of Bioactive Compounds from Latin American Plants. Supercritical Fluid Extraction of Nutraceuticals and Bioactive Compounds.

[38] Pereira C.G., Meireles M.A.A. (2010). Supercritical fluid extraction of bioactive compounds: Fundamentals, applications and economic perspectives. Food Bioprocess Technol..

[39] Mittal A., Sara U.V.S., Ali A., Aqil M. (2009). Status of fatty acids as skin penetration enhancers-A review. Curr. Drug Deliv..

[40] Guo H., Liu Z., Li J., Nie S., Pan W. (2006). Effects of isopropyl palmitate on the skin permeation of drugs. Biol. Pharm. Bull..

[41] Viljoen J.M., Cowley A., Du Preez J., Gerber M., du Plessis J. (2015). Penetration enhancing effects of selected natural oils utilized in topical dosage forms. Drug Dev. Ind. Pharm..

[42] Storey A., McArdle F., Friedmann P.S., Jackson M.J., Rhodes L.E. (2005). Eicosapentaenoic acid and docosahexaenoic acid reduce UVB- and TNFalpha- induced IL-8 secretion in keratinocytes and UVB-induced IL-8 in fibroblasts. J. Investig. Dermatol..

[43] Arantes E.L., Dragano N., Ramalho A., Vitorino D., de-Souza G.F., Lima M.H.M., Velloso L.C., Araújo E.P. (2016). Topical docosahexaenoic acid (DHA) accelerates skin wound healing in rats and activates GPR120. Biol. Res. Nurs..

[44] Álvarez-Viñas M., Flórez-Fernández N., Torres M.D., Domínguez H. (2019). Successful approaches for a red seaweed biorefinery. Mar. Drugs.

[45] Peñuela A., Robledo D., Bourgougnon N., Bedoux G., Hernández-Núñez E., Freile-Pelegrín Y. (2018). Environmentally friendly valorization of *Solieria filiformis* (Gigartinales, Rhodophyta) from IMTA using a biorefinery concept. Mar. Drugs.

[46] Rocha C.P., Pacheco D., Cotas J., Marques J.C., Pereira L., Ana M.M. (2021). Gonçalves. Seaweeds as Valuable Sources of Essential Fatty Acids for Human Nutrition. Int. J. Environ. Res. Public Health.

[47] Singleton V.L., Rossi J.A. (1965). Colorimetry of total phenolics with phosphomolybdic-phosphotungstic acid reagents. Am. J. Enol. Vitic..

[48] Bradford M.M. (1976). A rapid and sensitive method for the quantitation of microgram quantities of protein utilizing the principle of protein-dye Binding. Anal. Biochem..

[49] von Gadow A., Jounert E., Hansmann C.F. (1997). Comparison of the antioxidant activitiy aspalathin with that of other plant phenols of Rooibos tea (*Aspalathus linearis*), α-Tocopherol, BHT and BHA. J. Agric. Food Chem..

[50] Re R., Pellegrini N., Proteggente A., Pannala A., Yang M., Rice-Evans C. (1999). Antioxidant activity applying an improved ABTS radical cation decolorization assay. Free Radic. Biol. Med..

